# Auto-antibodies against interferons are common in people living with chronic hepatitis B virus infection and associate with PegIFNα non-response

**DOI:** 10.1016/j.jhepr.2025.101382

**Published:** 2025-02-28

**Authors:** Douglas L. Fink, David Etoori, Robert Hill, Orest Idilli, Nikita Kartikapallil, Olivia Payne, Sarah Griffith, Hannah F. Bradford, Claudia Mauri, Patrick T.F. Kennedy, Laura E. McCoy, Mala K. Maini, Upkar S. Gill

**Affiliations:** 1 Infection and Immunity, University College London, London, UK; 2 Royal Free London NHS Foundation Trust, London, UK; 3 Institute for Global Health, University College London, London, UK; 4 Barts Liver Centre, Blizard Institute, Barts and The London, School of Medicine & Dentistry, Queen Mary University of London, London, UK

**Keywords:** chronic hepatitis B, CHB, interferon, auto-antibodies

## Abstract

**Background & Aims:**

Type one (T1) and three interferons (T3IFNs) are implicated in chronic hepatitis B (CHB) immunopathogenesis. IFN remains the only licenced immune modulating therapy for CHB. We measured the prevalence of auto-antibodies (auto-Abs) against T1 and T3IFNs to examine the hypothesis that they impact HBV control and treatment response, as highlighted by COVID-19.

**Methods:**

Our multi-centre retrospective longitudinal study accessed two CHB cohorts; auto-Ab levels and neutralisation status were measured against T1IFN and T3IFN. Associations were tested against HBV clinical parameters.

**Results:**

Overall, 16.7% (46/276) of patients with CHB had any detectable anti-IFN auto-Abs at any time and 6.5% (18/276) anti-T3IFN auto-Abs, with a high incidence of PegIFNα-induced *de novo* auto-Abs (31.4%, 11/35). However, only a minority of auto-Ab-positive sera demonstrated neutralisation *in vitro* (4/46, 8.7%). Auto-Ab positivity correlated with higher median HBsAg levels (*p* = 0.0110). All individuals with detectable anti-T1IFN auto-Abs were PegIFNα non-responders.

**Conclusions:**

Non-neutralising anti-IFN auto-Abs are common in CHB and associate with higher median HBsAg levels. Further prospective study of anti-cytokine auto-Abs in CHB are required to characterise the association with long-term outcomes.

**Impact and implications:**

HBV and PegIFNα individually may induce broad autoreactivity associated with dysregulated antiviral immune responses. Auto-Ab screening prior to PegIFNα treatment or other immunotherapies may play a critical role in predicting treatment responses.

## Highlights

• Levels and prevalence of auto-Abs against three different antiviral IFN proteins were higher in patients with CHB.
• Auto-Abs against all three IFN subtypes developed *de novo* after PegIFNα therapy, suggesting induction of broad auto-reactivity.
• Despite high levels of anti-IFN auto-Abs, most did not lead to functional neutralisation of IFN *in vitro.*
• Individuals with any detectable anti-IFN auto-Abs had higher HBsAg levels, suggesting dysregulated antiviral responses.
• All individuals who responded to PegIFNα therapy had no detectable auto-Abs against type-1 IFN.

## Graphical abstract

**Figure:**
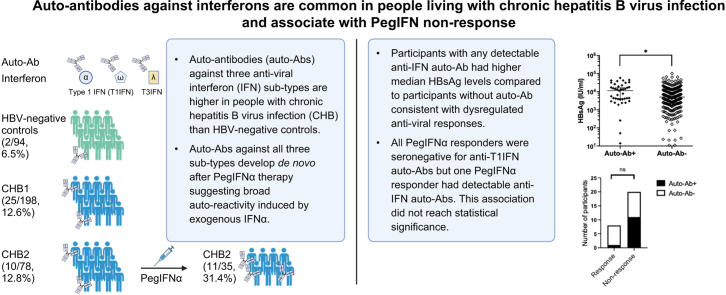

## Introduction

Chronic hepatitis B (CHB) causes major global morbidity and mortality.^1^ Functional cure, defined as sustained HBsAg clearance, following a period of antiviral therapy is a rare event.^2^ Understanding the immunobiology of naturally resolving infection is key to developing immunotherapies for HBV cure. HBV clearance depends on coordination of innate and adaptive immunity, including IFN signalling.^3^^,^^4^ Of three IFN subtypes, T1 and T3IFNs are most strongly associated with antiviral responses through IFN-stimulated gene regulation.^5^^,^^6^ The role of the diverse IFN signalling pathways in CHB immunopathogenesis remains poorly understood. Analyses of peripheral blood have suggested that IFN secretion is limited during HBV infection.[7], [8], [9], [10] Recent transcriptomic analyses of liver tissue, however, suggest that IFN-stimulated gene expression correlates with liver inflammation.[11], [12], [13] Equivalent tissue-level analyses for T3IFN are lacking, but T3IFN is secreted by HBV-infected hepatocytes with antiviral properties *in vitro*.^14^^,^^15^

Recent studies have established the dramatic effect on mortality of acquired auto-Abs neutralising T1IFN in COVID-19.^16^ This has prompted re-evaluation of auto-Abs in other infectious diseases. In CHB the impact of anti-IFN auto-Abs remains uncertain in the natural history of infection and in determining treatment outcomes. In COVID-19, auto-Abs are predominantly against T1IFNs IFNα and IFNω but there are no studies of anti-IFNω or anti-T3IFN auto-Ab in CHB.

Exogenous IFNα is used as a therapy in CHB, with a finite course. Polyethylene glycol-conjugated (‘pegylated’) derivates of IFNα (PegIFNα) were introduced in 2002 to improve pharmacokinetics. Prior to PegIFNα, 7-39% of IFNα-treated individuals were estimated to develop anti-IFNα auto-Abs which associated with non-response.^17^ In the PegIFNα era, a single study suggested that up to 47% of individuals with any prior history of IFN therapy had detectable anti-IFNα auto-Abs.^18^ The study found no association with treatment response but did not provide methodology for defining serology assay cut-off values, nor a measure of auto-Ab function *in vitro*. Importantly HBsAg clearance rates post-PegIFNα are low but mechanisms of CHB insensitivity *in vivo* to IFNα remain uncertain.^19^^,^^20^ This knowledge gap is particularly relevant while IFNs continue to be included in trials aimed at HBV and HDV cure where anti-IFN auto-Abs may compromise outcomes.^17^^,^^21^

We measured the prevalence and function of auto-Abs against two subtypes of T1IFN (IFNα and IFNω) and against T3IFN (IFNλ1) in two cohorts of people living with CHB, including longitudinal samples in individuals receiving PegIFNα. We tested for associations between auto-Ab and clinical parameters.

## Patients and methods

### Study population and clinical metadata

We retrospectively assayed cryopreserved sera from two CHB cohorts undergoing routine follow-up (CHB1 [n = 198] from Central and North West London NHS Foundation Trust, University College London Hospitals NHS Foundation Trust and Royal Free London NHS Foundation Trust (RFL); CHB2 [n = 78] from Royal London Hospital, Barts Health NHS Trust including individuals [n = 36] receiving PegIFNα therapy). CHB1 participants were significantly older with HBeAg-negative disease (Table S1). PegIFNα treatment responses were defined in accordance with international guidelines.^22^ All clinical outcomes were obtained during routine appointments. HBV-uninfected healthy controls (n = 94) were recruited from university research and hospital clinical staff. Samples from patients with systemic lupus erythematosus, with known anti-T1IFN auto-Ab, were used as positive controls.^23^

### Anti-IFN auto-Ab detection

Anti-IFN IgG detection was undertaken using Gyrolab microfluidic immunoassay platform as previously described with wash, capture (recombinant IFN) and detect (anti-human IgG Ab) reagents and 1:10 PBS-diluted samples.^24^ Results are expressed as semi-quantitative arbitrary units. The detection cut-off was defined as 2 standard deviations below the mean healthy control values.

### IFN signalling bioassay

To assess the ability of patient serum to block T1IFN and T3IFN pathway activation, HEK-293 cells were used which express luciferase, controlled by an *ISRE* (IFN-sensitive response element) sensitive to both T1IFN and T3IFN signalling.^25^ HEK-293-*ISRE* cells were cultured at 37 °C 5% CO_2_ in DMEM containing 10% FBS and 1% penicillin/streptomycin. Assays were performed in photometric 96 well-plates with cells seeded at a density of 2x10^5^ cells/ml. Serum was used with an end dilution of 1:10 in the presence of media (no IFN), 0.1 ng/ml IFNα2a (hereafter IFNα), 0.1 ng/ml IFNω, or 5 ng/ml IFNλ1 (hereafter IFNλ). The cells were incubated overnight, cells lysed, luciferase substrate added, and light units measured using a luminometer (BioTek Synergy H1 Multimode Reader). Samples were run in duplicate on different plates. IFN signalling activity was calculated from fold induction of samples over negative control (media only) and expressed as percentages normalised to activation by each IFN dose.

### Statistical analyses

All analyses were performed using GraphPad Prism V10.0.2 or STATA. Mann-Whitney *U* test or Kruskal-Wallis test (for groups of ≥3) were used to compare unpaired data, while Wilcoxon matched-pairs sign rank test (Wilcoxon) was used to compare paired data. Fisher’s exact test or Chi-squared test were used for contingency tables. Auto-Ab levels were log transformed as continuous dependent variables to fit a random intercepts model which accounted for clustering by individuals. Separate models were run for each auto-Ab. Given the exploratory nature of the study, univariate analysis was performed for *a priori* variables. For CHB1, missing data were interpreted as missing completely at random and no substitution or imputation methods were applied. *p* values <0.05 were considered statistically significant.

### Ethics

Serum samples were accessed via RFL and UCL Biobank ethical Review Committee (UCL/RFL Biobank; REC reference: 11/WA/0077 or 21/WA/0388), Barts and The London NHS Trust Ethics Review Board (REC reference 10/H0715/39 or 16/LO/1699) and UCL systemic lupus erythematosus cohort study (REC reference no. 14/SC/1200). Informed consent was obtained from all participants.

## Results

**Fig. 1:**
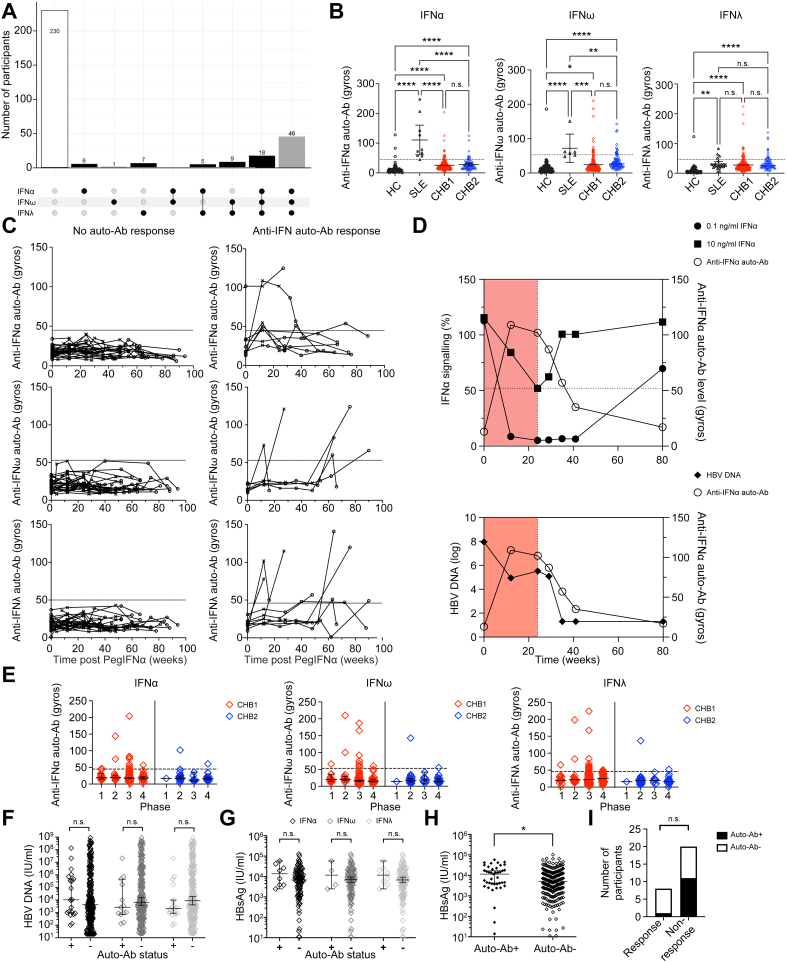
Prevalence, function and clinical associations of auto-Abs against three subtypes of IFN. (A) Upset plot of number of participants with serum auto-Ab below (hollow bars) and above the level of detection (solid bars) measured by Gyros immunoassay, against three IFN subtypes with intersecting seroreactivity illustrated by upset plot connecting lines. Participants with any auto-Ab at any time are shown by the grey bar. (B) Scatter plot of serum auto-Ab levels against three IFN subtypes expressed as arbitrary units (Gyros). HC (healthy controls), SLE (patients with SLE and known auto-Ab status), CHB1, CHB2 (highest auto-Ab levels from longitudinal samples shown for CHB2). The cut-off for detection for each auto-Ab is shown by the dotted horizontal line. Median levels shown with error bars for 95% CIs. Analysed by Kruskall-Wallis ANOVA with Bonferroni correction. (C) Line charts for serum auto-Ab levels against three IFN subtypes in longitudinal CHB2 samples 2012-2016 for individuals with no and any auto-Ab positivity. Current PegIFNα treatment is indicated by cross plots. (D) IFNα signalling neutralisation by single participant longitudinal serum samples, with anti-IFNα auto-Ab levels (empty circle plots) plotted against 0.1 ng/ml IFNα (solid circle plots) and 10 ng/ml IFNα (square plots) signalling activation, or HBV DNA (log). Cut-off of detection for auto-Ab shown by horizontal dotted line. PegIFNα exposure shown by pink block prior to NUC therapy. (E) Auto-Ab levels against three IFN subtypes organised by CHB phase for CHB1 and CHB2 without PegIFNα exposure. (F) HBV DNA (IU/ml; n = 36 missing data) and (G) HBsAg levels (IU/ml; n = 44 missing data) for all individuals with CHB without PegIFNα exposure organised by auto-Ab status against three IFN subtypes. (H) HBsAg levels for all CHB (with or without PegIFNα exposure) organised into columns with or without any detectable auto-Ab. For (E-H), median levels shown with error bars for 95% CIs. Analysed by Mann-Whitney *U* test. (I) Frequency of auto-Abs by PegIFNα response and non-response. Analysed by Fisher’s exact test. n.s. = not significant; ∗*p <*0.05, ∗∗*p <*0.05,∗∗∗∗*p <*0.0001. Auto-Abs, auto-antibodies; CHB, chronic hepatitis B; HC, healthy control; IFN, interferon; NUC, nucleos(t)ide analogue; PegIFNα, pegylated-IFNα; SLE, systemic lupus erythematosus.

Overall, 16.7% (46/276) of patients with CHB showed evidence of any anti-IFN auto-Ab production, with 6.5% (18/276) demonstrating auto-Abs against all three IFN subtypes (Table 1; Fig. 1A). Compared to healthy controls, people with CHB from either cohort had a higher frequency of detectable auto-Abs against IFNα (10.5% *vs.* 3.2%, *p* = 0.0324; Table 1; Fig. 1B), IFNω (10.1% *vs*. 1.1%, *p* = 0.0030) and IFNλ (14.1% *vs.* 1.1%, *p <*0.0001). Median auto-Ab levels against all IFN subtypes were also significantly higher in both CHB cohorts compared to healthy controls (Fig. 1B).

**Table 1:** Overall anti-IFN auto-Ab outcomes including PegIFNα-exposed participants.

	Healthy controls	CHB1	CHB2	CHB total
Total	94	198	78	276
Anti-IFNα auto-Ab positive (n,%)	3 (3.2)	18 (9.1)	11 (14.1)	29 (10.5)
Median level (Gyros; 95% CI)	8 (6-10)	20 (18-22)	16 (15-21)	-
*De novo* during PegIFNα	-	-	7	-
Neutralising	0	0	1	1
Anti-IFN*ω* auto-Ab positive	1 (1.1)	16 (8.1)	12 (15.3)	28 (10.1)
Median level	9 (8-11)	17 (15-19)	16 (14-22)	-
*De novo* during PegIFNα	-	-	7	-
Neutralising	0	2	0	2
Anti-IFN*λ* auto-Ab positive	1 (3.2)	24 (12.1)	15 (19.2)	39 (14.1)
Median level	15 (10-18)	21 (19-24)	17 (15-21)	-
*De novo* during PegIFNα	-	-	9	-
Neutralising	0	0	0	0
Any anti-T1IFN auto-Ab positive	2 (6.5)	21 (10.6)	18 (23.1)	39 (14.1)
*De novo* during PegIFNα	-	-	11	-
Neutralising	0	2	1	3
Any auto-Ab	2 (6.5)	25 (12.6)	21 (26.9)	46 (16.7)
*De novo* during PegIFNα	-	-	11	-
Neutralising	0	2	1	3

Auto-Abs, auto-antibodies; CHB, chronic hepatitis B; IFN, interferon; PegIFNα, pegylated-IFNα.

For participants in CHB2 with serum samples available pre- and post-PegIFNα therapy, 20.0% (7/35) developed *de novo* anti-IFNα auto-Abs, 20.0% (7/35) anti-IFNω auto-Abs, and 25.7% (9/35) anti-IFNλ auto-Abs (Table 1; Fig. 1C). There were no clear differences in hepatology parameters prior to initiation of PegIFNα therapy between those who developed *de novo* auto-Abs compared to those who did not (Table S2). Individuals (n = 7) developing *de novo* anti-IFNα auto-Abs after PegIFNα treatment sero-reverted within 12 months and auto-Ab levels continued to increase post-PegIFNα therapy in one individual (Fig. 1C). Most individuals acquiring auto-Abs against IFNω and IFNλ remained seropositive throughout the course of follow-up (Fig. 1C).

We tested IFN neutralisation *in vitro* and showed this was only present in one individual with anti-IFNα auto-Abs (1/29, 3.4%; Fig. 1D), while receiving PegIFNα, and three individuals with anti-IFNω auto-Abs (3/28, 10.7%), one with and two without PegIFNα exposure (Table S1).

There was no difference in auto-Ab levels between CHB phases (Fig. 1E) and median levels of HBV DNA in treatment-naïve individuals did not significantly differ by auto-Ab status (Fig. 1F). Median levels of HBsAg were higher for individuals with detectable auto-Abs against each IFN subtype compared to individuals without auto-Abs, although these differences did not individually reach statistical significance (Fig. 1G). Overall median HBsAg levels were greater in patients with any detectable auto-Abs (11,466 IU/ml, 95% CI 4,236-16,655) compared to those without (5,076 IU/ml, 95% CI 4,119-6,609, *p* = 0.0110; Fig. 1H). A significant association between anti-IFNα auto-Abs and HBsAg levels was noted by random intercepts modelling (coefficient 0.11, 95% CI 0.02-0.19, *p* = 0.017; Table S3). Interestingly, all individuals with any detectable anti-T1FN auto-Abs demonstrated non-response to PegIFNα. All PegIFNα responders were seronegative for anti-T1IFN auto-Abs but one PegIFNα responder had detectable anti-IFNλ auto-Abs (Fig. 1I); however, this association within a small cohort did not reach statistical significance (*p* = 0.0882).

## Discussion

In this study, we demonstrate the high prevalence of auto-Abs against T1 and T3IFNs in people living with CHB; these auto-Abs were associated with impaired control of HBV replication and non-response to PegIFNα treatment. We noted, in our study, that the incidence of developing anti-IFNα auto-Abs, induced by PegIFNα, was comparable to that seen with standard IFN prior to pegylation (7-39%).^17^ Most participants with auto-Abs had reactivities against two or three IFN subtypes (32/46, 69.6%). Co-carriage of anti-IFNα and anti-IFNω auto-Abs is as high as 51% in COVID-19,^26^^,^^27^ and anti-T3IFN auto-Ab prevalence has only been measured previously in two COVID-19 studies (3.6-10.1%)^28^^,^^29^ and thus prior data in CHB remains limited; our study is thus key in filling this knowledge gap.

The prevalence of neutralising auto-Abs in our study (4/276, 1.4%) remained low, but was notably still greater than reported in the general population.^28^^,^^30^ Most binding auto-Abs did not neutralise their target antigen *in vitro,* for which there may be various reasons. Potential insensitivity of the reporter assay is a possibility, although this has been used across other studies^30^ or there may be alternative non-Fab-mediated effector functions of auto-Abs *in vivo*. Importantly for pathogenic anti-IFN γ auto-Abs, associated with severe mycobacterial and fungal infections, Fc-mediated antagonism of T1IFN signalling and innate cell cytotoxicity have been reported.^31^ Thus, the effector function of auto-Abs detected in our study may not be Fab-mediated, potentially explaining the apparent association with increased virus replication.

Even in the absence of specific anti-IFNα auto-Abs, all individuals with any auto-Abs demonstrated PegIFNα non-response. Carriage of any auto-Ab was associated with higher median HBsAg levels, consistent with the hypothesis that these auto-Abs may antagonise antiviral IFN signalling, leading to compromised HBV control. Conversely, high HBsAg load may drive the development of anti-IFNα auto-Abs, due to the potential induction of IFNα during spontaneous hepatic flares^32^ There is minimal structural homology between T1 and T3IFNs, which suggests that the presence of auto-Abs against multiple IFN subtypes, rather than evidence of cross-reactivity, represents broad breaches of immune tolerance as seen in COVID-19. PegIFNα therapy is also associated with *de novo* anti-thyroid auto-Abs.^33^ Auto-Abs may therefore represent biomarkers reflecting an immune phenomenon predisposing to increased HBV replication that is IFN-refractory, such as increased frequency of atypical B cells which are associated with auto-Ab production and impaired anti-HBV immunity.^34^^,^^35^

The major limitation of our study is its retrospective design; thus, the sampling strategy was not designed to support definitive analyses for auto-Ab seroprevalence or associations with clinical outcomes. The relationship between HBsAg and auto-Ab is intriguing, however, we are unable to infer direction of cause or effect. Despite longitudinal sampling, auto-Abs were frequently only detected late in follow-up, which limits characterisation of the trajectory of anti-IFN auto-Ab production. Furthermore, due to the retrospective study design, we acknowledge some missing data, and although no CHB1 participants were receiving PegIFNα at the time of sample collection, we cannot exclude historical IFN therapy. Previous IFN treatment in some CHB1 participants may explain why overall auto-Ab profiles were comparable between the two cohorts.

In summary, our study suggests that anti-T1 and T3IFN auto-Abs are common in the UK CHB population. Our data can be used to design larger seroprevalence and immunophenotyping studies to characterise this and broader autoreactivity in CHB, which may be associated with treatment failure and have significant implications for future immune-mediated HBV therapies. PegIFNα-induced neutralising auto-Abs could be risk factors for life-threatening outcomes of acute respiratory virus infection and live-virus vaccinations in the CHB population which may require enhanced monitoring.

## Abbreviations

Auto-Abs, auto-antibodies; CHB, chronic hepatitis B; IFN, interferon; PegIFNα, pegylated-IFNα; T1IFN, type 1 IFN; T3IFN, type 3 IFN.

## Financial support

This works was partly supported by a University College London (UCL) Therapeutic Acceleration Scheme Grant (553191.D-OTH.178973).

## Authors’ contributions

Conceived and designed study, performed some experiments and analysed data, and wrote the manuscript: DLF. Designed study, analysed data and wrote the manuscript: USG, MKM. Provided intellectual input and resources: HFB, MC, PTFK, LEM. Analysed data and performed statistical model analyses: DE. Performed experiments and analysed data: RH, OI, NK, OP, SG. Critically reviewed and approved the final manuscript: all authors.

## Data availability statement

The data supporting the results of this study are available from the corresponding author upon reasonable request. The data are not publicly available due to privacy/ethical restrictions.

## Conflicts of interest

MKM has received collaborative research funding from Gilead Sciences, F. Hoffmann-La Roche and Immunocore and has served as a consultant or on advisory boards for Gilead Sciences, F. Hoffmann-La Roche, Immunocore and GSK. Other authors have no conflicts of interest.

Please refer to the accompanying ICMJE disclosure forms for further details.
