# Corrigendum to “Auto-antibodies against interferons are common in people living with chronic hepatitis B virus infection and associate with PegIFNα non-response” [J Hepatol (2025) 101382]

**DOI:** 10.1016/j.jhepr.2025.101520

**Published:** 2025-08-22

**Authors:** Douglas L. Fink, David Etoori, Robert Hill, Orest Idilli, Nikita Kartikapallil, Olivia Payne, Sarah Griffith, Hannah F. Bradford, Claudia Mauri, Patrick T.F. Kennedy, Laura E. McCoy, Mala K. Maini, Upkar S. Gill

**Affiliations:** 1 Infection and Immunity, University College London, London, UK; 2 Royal Free London NHS Foundation Trust, London, UK; 3 Institute for Global Health, University College London, London, UK; 4 Barts Liver Centre, Blizard Institute, Barts and The London, School of Medicine & Dentistry, Queen Mary University of London, London, UK

It has come to our attention that key information was missing from the ‘Financial support’ statement in our manuscript. This statement should read: ‘This work was partly supported by a University College London (UCL) Therapeutic Acceleration Scheme Grant (553191.D-OTH.178973). The funds for this grant were provided by the Wellcome Trust.’ We apologise for any inconvenience caused.

