# Supplementary material for: Auto-antibodies against interferons are common in people living with chronic hepatitis B virus infection and associate with PegIFNα non-response
Source: JHEP Rep. 2025 Feb 28;7(5):101382. doi: 10.1016/j.jhepr.2025.101382 (PMC12018104; doi:10.1016/j.jhepr.2025.101382)
Supplement: Multimedia component 1 [file mmc1.pdf]

Auto-antibodies against interferons are common in people living with chronic hepatitis B virus infection and associate with PegIFNα non-response

Douglas L. Fink, David Etoori, Robert Hill, Orest Idilli, Nikita Kartikapallil, Olivia Payne, Sarah Griffith, Hannah F. Bradford, Claudia Mauri, Patrick T.F. Kennedy, Laura E. McCoy, Mala K. Maini, Upkar S. Gill

Table S1……………………………………………………………………………………2

Table S2……………………………………………………………………………………3

Table S3……………………………………………………………………………………4

	HC	CHB1	CHB2	p value
Total	94	198	78	
Age (IQR)	40 (31-64)	48 (41-54)	32 (28-41)	0.0001
Sex<br>Male<br>Female<br>Missing	 47 (50.0)<br>44 (46.8)<br>3 (3.2)	 102 (51.5)<br>75 (37.9)<br>21 (10.6)	-	-
HBeAg<br>Positive<br>Negative<br>Missing	-	 30 (15.2)<br>139 (70.2)<br>29 (14.6)	 36 (46.1)<br>41 (52.6)<br>1 (1.3)	<0.001
HBV DNA<br>Undetectable<br>Detectable<br>Missing	-	 29 (14.6)<br>134 (67.7)<br>35 (17.7)	 6 (7.7)<br>71 (91.0)<br>1 (1.3)	0.04
Median log HBV DNA<br>(IU/ml, IQR)	-	3.17<br>(2.00-4.14)	6.07<br>(3.77-7.54)	<0.001
Median log HBsAg<br>(IU, IQR)<br>Missing	-	3.59<br>(3.08-4.27)<br>43 (21.7)	3.91<br>(3.52-4.19)<br>1 (1.3)	0.1229
Median ALT<br>(IU/L, IQR)<br>Missing	-	33<br>(23-48)<br>34 (17.2)	65<br>(35-129)<br>1 (1.3)	<0.001
CHB phase<br>1<br>2<br>3<br>4<br>5<br>Unknown	-	 14 (7.1)<br>15 (7.6)<br>108 (54.5)<br>26 (13.1)<br>2 (1.0)<br>33 (16.7)	 8 (10.3)<br>28 (35.9)<br>14 (17.9)<br>27 (34.6)<br>0<br>1 (1.3)	<0.001
PegIFNα exposure<br>Samples pre and post PegIFNα	-	0<br>0	58 (74.3)<br>35 (44.8)	-
Nucleoside treatment during study	-	26 (13.1)	35 (44.8)	<0.001

Table S1. Cohort clinical characteristics.

HC= healthy controls ; CHB1= chronic hepatitis B virus infection cohort 1; CHB2= chronic hepatitis B virus cohort 2 (without PegIFNα exposure); IQR=inter-quartile range; ALT=alanine transaminase. Continuous variables reported for first available sample for CHB2 cohort with longitudinal sampling. Comparisons of median values by Mann-Whitney U-test or Kruskal-Wallis test (for groups of 3 or more); comparisons of frequency by Chi-squared test or Fisher's exact test (for groups of 5 or less). $p<0.05$ = statistically significant.

	No auto-Ab	Anti-IFNα auto-Ab	Anti-IFNω auto-Ab	Anti-IFNλ auto-Ab	P value
Total<br>Auto-Ab pre PegIFNα<br>Auto-Ab post PegIFNα	22 (61.1)<br>-<br>-	8 (22.2)<br>1<br>7	7 (19.4)<br>0<br>7	9 (25.0)<br>0<br>9	-
Age (IQR)	34 (29-43)	35 (29-43)	38 (29-45)	35 (28-45)	0.7256
HBeAg<br>Positive<br>Negative<br>Missing	 10 (45.5)<br>12<br>0	 4 (50.0)<br>4<br>0	 1 (14.3)<br>5<br>1	 3<br>6<br>0	0.6024
Median log HBV DNA pre PegIFNα (IU/ml, IQR)	6.6 (6.1-7.7)	8.1 (6.4-8.6)	6.4 (4.6-8.1)	6.9 (4.8-8.1)	0.2799
Median log HBsAg pre PegIFNα (IU, IQR)	3.9 (3.8-4.2)	4.0 (3.1-4.6)	4.1 (3.9-4.3)	4.0 (3.8-4.3)	0.5144
Median ALT pre PegIFNα (IU/L, IQR)	114 (83-256)	164 (73-210)	97 (57-210)	91 (61-199)	0.8743
Median Ishak liver fibrosis score (IQR)	3.0 (1.0-4.0)	3.0 (1.0-3.5)	1.5 (1.0-2.5)	2.0 (1.0-3.0)	0.6693
Median HAI score (IQR)	5.0 (4.0-8.0)	6.0 (3.5-7.0)	5.0 (3.5-6.3)	5.0 (4.0-7.0)	0.9285
HBV genotype<br>A<br>B<br>C<br>D<br>E<br>Missing	 2<br>1<br>4<br>8<br>6<br>2	 1<br>0<br>1<br>3<br>1<br>1	 1<br>0<br>0<br>1<br>2<br>3	 0<br>0<br>1<br>1<br>3<br>3	0.7390

Table S2. Clinical characteristics of CHB2 PegIFNα-exposed individuals with serum samples pre and post PegIFNα

HC= healthy controls ; CHB1= chronic hepatitis B virus infection cohort 1; CHB2= chronic hepatitis B virus cohort 2 (without PegIFNα exposure); IQR=inter-quartile range; ALT=alanine transaminase; HAI=Histological Activity Index. Comparisons of median values by Mann-Whitney U-test or Kruskal-Wallis test (for groups of 3 or more); comparisons of frequency by Chi-squared test or Fisher's exact test (for groups of 5 or less). $p<0.05$ = statistically significant.

		IFNα		IFNω		IFNλ	
Total	513						
	Median [IQR]	Coefficient (95% CI)	p-value	Coefficient (95% CI)	p-value	Coefficient (95% CI)	p-value
log(HBsAg)	3.73 [3.37, 4.14]	**0.11 (0.02, 0.19)**	**0.017**	0.02 (-0.07, 0.11)	0.64	0.02 (-0.07, 0.12)	0.606
log(HBVDNA)	3.08 [1.46, 5.19]	0.01 (-0.01, 0.03)	0.181	0.01 (-0.01, 0.03)	0.429	-0.003 (-0.04, 0.02)	0.805
log(ALT)	1.59 [1.40, 1.85]	0.03 (-0.07, 0.12)	0.566	-0.003 (-0.12, 0.11)	0.958	-0.06 (-0.19, 0.07)	0.366
	n (%)						
Age							
16-24	25 (4.9)	-0.02 (-0.32, 0.27)	0.876	-0.06 (-0.39, 0.28)	0.743	**-0.42 (-0.77, -0.08)**	**0.017**
25-34	161 (31.4)	Reference	__	Reference	__	Reference	__
35-44	150 (29.2)	0.03 (-0.13, 0.19)	0.721	0.002 (-0.17, 0.17)	0.983	0.07 (-0.10, 0.24)	0.432
45-54	117 (22.8)	0.13 (-0.05, 0.31)	0.159	0.17 (-0.01, 0.35)	0.063	0.17 (-0.02, 0.35)	0.077
55+	57 (11.1)	0.04 (-0.16, 0.25)	0.676	-0.03 (-0.24, 0.18)	0.79	0.09 (-0.13, 0.30)	0.42
HBeAg							
Negative	328 (63.9)	Reference	__	Reference	__	Reference	__
Positive	148 (28.9)	0.07 (-0.06, 0.20)	0.278	0.12 (-0.02, 0.26)	0.09	0.01 (-0.13, 0.16)	0.864
Missing	37 (7.2)	__	__	__	__	__	__
IFN therapy at time of sample							
No IFN exposure	268 (52.2)	Reference	__	Reference	__	Reference	__
Current IFN exposure	81 (15.8)	**0.17 (0.06, 0.27)**	**0.002**	**0.14 (0.01, 0.27)**	**0.031**	0.12 (-0.02, 0.27)	0.098
Post IFN exposure	158 (30.8)	0.01 (-0.09, 0.11)	0.83	**0.16 (0.04, 0.28)**	**0.011**	0.09 (-0.04, 0.23)	0.161

Table S3. Random intercepts model for auto-Ab levels

Random intercepts model for log transformed auto-Ab levels against IFN sub-types organised by univariate analyses for all available CHB samples from both cohorts including longitudinal samples (n=513). Significant interactions are highlighted in bold where $p < 0.05$.
