# Supplementary material for: Auto-antibodies against interferons are common in people living with chronic hepatitis B virus infection and associate with PegIFNα non-response
Source: JHEP Rep. 2025 Feb 28;7(5):101382. doi: 10.1016/j.jhepr.2025.101382 (PMC12018104; doi:10.1016/j.jhepr.2025.101382)
Supplement: Multimedia component 2 [file mmc2.docx]

**JHEP Reports**

**CTAT methods**

Tables for a “Complete, Transparent, Accurate and Timely account” (CTAT) are now mandatory for all revised submissions. The aim is to enhance the reproducibility of methods.

- Only include the parts relevant to your study
- Refer to the CTAT in the main text as ‘Supplementary CTAT Table’
- Do not add subheadings
- Add as many rows as needed to include all information
- Only include one item per row

**If the CTAT form is not relevant to your study, please outline the reasons why:**

|  |
| --- |

- 1. **Antibodies**

| **Name** | **Citation** | **Supplier** | **Cat no.** | **Clone no.** |
| --- | --- | --- | --- | --- |
|  |  |  |  |  |

- 1. **Cell lines**

| **Name** | **Citation** | **Supplier** | **Cat no.** | **Passage no.** | **Authentication test method** |
| --- | --- | --- | --- | --- | --- |
| Lucia luciferase HEK293 cells | PMID:35237663 | InvivoGen | Hkl-null | 20 | ISG PCR |

- 1. **Organisms**

| **Name** | **Citation** | **Supplier** | **Strain** | **Sex** | **Age** | **Overall n number** |
| --- | --- | --- | --- | --- | --- | --- |
|  |  |  |  |  |  |  |

- 1. **Sequence based reagents**

| **Name** | **Sequence** | **Supplier** |
| --- | --- | --- |
|  |  |  |

- 1. **Biological samples**

| **Description** | **Source** | **Identifier** |
| --- | --- | --- |
| Serum | Central and North West London NHS Foundation Trust, University College London Hospitals NHS Foundation Trust and Royal Free London NHS Foundation Trust | CHB1 |
| **Serum** | Royal London Hospital, Barts Health NHS Trust | CHB2 |
| **Serum** | University College London Hospitals NHS Foundation Trust | SLE |
| **Serum** | Infection and Immunity, UCL, and Royal Free London NHS Foundation Trust | HC |

- 1. **Deposited data**

| **Name of repository** | **Identifier** | **Link** |
| --- | --- | --- |
|  |  |  |

- 1. **Software**

| **Software name** | **Manufacturer** | **Version** |
| --- | --- | --- |
| Prism | GraphPad | 10 |

- 1. **Other (*e.g*. drugs, proteins, vectors etc.)**

| Gyrolab xPlore | Gyros Protein Technologies | 1 |
| --- | --- | --- |
|  |  |  |

- 1. **Please provide the details of the corresponding methods author for the manuscript:**

| **Douglas Fink, UCL Institute of Immunity and Transplantation, Pears building, Pond street, NW3 2PP, London, UK; +442077940500; d.fink@ucl.ac.uk** |
| --- |

**2.0 Please confirm for randomised controlled trials all versions of the clinical protocol are included in the submission. These will be published online as supplementary information.**

| **NA** |
| --- |
