# Supplementary material for: Auto-antibodies against interferons are common in people living with chronic hepatitis B virus infection and associate with PegIFNα non-response
Source: JHEP Rep. 2025 Feb 28;7(5):101382. doi: 10.1016/j.jhepr.2025.101382 (PMC12018104; doi:10.1016/j.jhepr.2025.101382)
Supplement: Multimedia component 4 [file mmc4.pdf]

Auto-antibodies against interferons are common in people living with chronic hepatitis B virus infection and associate with PegIFNα non-response

Douglas L. Fink[1,2,*], David Etoori[3], Robert Hill[1], Orest Idilli[1], Nikita Kartikapallil[1], Olivia Payne[1], Sarah Griffith[1], Hannah F. Bradford[1], Claudia Mauri[1], Patrick T.F. Kennedy[4], Laura E. McCoy[1], Mala K. Maini[1], Upkar S. Gill[4]

JHEP Reports **2025**. vol. 7 | 1–6

Check for updates

Background & Aims: Type one (T1) and three interferons (T3IFNs) are implicated in chronic hepatitis B (CHB) immunopathogenesis. IFN remains the only licenced immune modulating therapy for CHB. We measured the prevalence of auto-antibodies (auto-Abs) against T1 and T3IFNs to examine the hypothesis that they impact HBV control and treatment response, as highlighted by COVID-19.

Methods: Our multi-centre retrospective longitudinal study accessed two CHB cohorts; auto-Ab levels and neutralisation status were measured against T1IFN and T3IFN. Associations were tested against HBV clinical parameters.

Results: Overall, 16.7% (46/276) of patients with CHB had any detectable anti-IFN auto-Abs at any time and 6.5% (18/276) anti-T3IFN auto-Abs, with a high incidence of PegIFNα-induced *de novo* auto-Abs (31.4%, 11/35). However, only a minority of auto-Ab-positive sera demonstrated neutralisation *in vitro* (4/46, 8.7%). Auto-Ab positivity correlated with higher median HBsAg levels (p = 0.0110). All individuals with detectable anti-T1IFN auto-Abs were PegIFNα non-responders.

Conclusions: Non-neutralising anti-IFN auto-Abs are common in CHB and associate with higher median HBsAg levels. Further prospective study of anti-cytokine auto-Abs in CHB are required to characterise the association with long-term outcomes.

© 2025 The Authors. Published by Elsevier B.V. on behalf of European Association for the Study of the Liver (EASL). This is an open access article under the CC BY-NC-ND license (http://creativecommons.org/licenses/by-nc-nd/4.0/).

Introduction

Chronic hepatitis B (CHB) causes major global morbidity and mortality.[1] Functional cure, defined as sustained HBsAg clearance, following a period of antiviral therapy is a rare event.[2] Understanding the immunobiology of naturally resolving infection is key to developing immunotherapies for HBV cure. HBV clearance depends on coordination of innate and adaptive immunity, including IFN signalling.[3,4] Of three IFN subtypes, T1 and T3IFNs are most strongly associated with antiviral responses through IFN-stimulated gene regulation.[5,6] The role of the diverse IFN signalling pathways in CHB immunopathogenesis remains poorly understood. Analyses of peripheral blood have suggested that IFN secretion is limited during HBV infection.[7–10] Recent transcriptomic analyses of liver tissue, however, suggest that IFN-stimulated gene expression correlates with liver inflammation.[11–13] Equivalent tissue-level analyses for T3IFN are lacking, but T3IFN is secreted by HBV-infected hepatocytes with antiviral properties *in vitro*.[14,15]

Recent studies have established the dramatic effect on mortality of acquired auto-Abs neutralising T1IFN in COVID-19.[16] This has prompted re-evaluation of auto-Abs in other infectious diseases. In CHB the impact of anti-IFN auto-Abs remains uncertain in the natural history of infection and in determining treatment outcomes. In COVID-19, auto-Abs are predominantly against T1IFNs IFNα and IFNω but there are no studies of anti-IFNω or anti-T3IFN auto-Ab in CHB.

Exogenous IFNα is used as a therapy in CHB, with a finite course. Polyethylene glycol-conjugated (‘pegylated’) derivates of IFNα (PegIFNα) were introduced in 2002 to improve pharmacokinetics. Prior to PegIFNα, 7-39% of IFNα-treated individuals were estimated to develop anti-IFNα auto-Abs which associated with non-response.[17] In the PegIFNα era, a single study suggested that up to 47% of individuals with any prior history of IFN therapy had detectable anti-IFNα auto-Abs.[18] The study found no association with treatment response but did not provide methodology for defining serology assay cut-off values, nor a measure of auto-Ab function *in vitro*. Importantly HBsAg clearance rates post-PegIFNα are low but mechanisms of CHB insensitivity *in vivo* to IFNα remain uncertain.[19,20] This knowledge gap is particularly relevant while IFNs continue to be included in trials aimed at HBV and HDV cure where anti-IFN auto-Abs may compromise outcomes.[17,21]

We measured the prevalence and function of auto-Abs against two subtypes of T1IFN (IFNα and IFNω) and against T3IFN (IFNλ1) in two cohorts of people living with CHB, including

* Corresponding author. Address: UCL Institute of Immunity and Transplantation, Pears building, Pond street, London, NW3 2PP, UK.
E-mail address: d.fink@ucl.ac.uk (D.L. Fink).
https://doi.org/10.1016/j.jhepr.2025.101382

longitudinal samples in individuals receiving PegIFNα. We tested for associations between auto-Ab and clinical parameters.

Patients and methods

Study population and clinical metadata

We retrospectively assayed cryopreserved sera from two CHB cohorts undergoing routine follow-up (CHB1 [n = 198] from Central and North West London NHS Foundation Trust, University College London Hospitals NHS Foundation Trust and Royal Free London NHS Foundation Trust (RFL); CHB2 [n = 78] from Royal London Hospital, Barts Health NHS Trust including individuals [n = 36] receiving PegIFNα therapy). CHB1 participants were significantly older with HBeAg-negative disease (Table S1). PegIFNα treatment responses were defined in accordance with international guidelines.[22] All clinical outcomes were obtained during routine appointments. HBV-uninfected healthy controls (n = 94) were recruited from university research and hospital clinical staff. Samples from patients with systemic lupus erythematosus, with known anti-T1IFN auto-Ab, were used as positive controls.[23]

Anti-IFN auto-Ab detection

Anti-IFN IgG detection was undertaken using Gyrolab microfluidic immunoassay platform as previously described with wash, capture (recombinant IFN) and detect (anti-human IgG Ab) reagents and 1:10 PBS-diluted samples.[24] Results are expressed as semi-quantitative arbitrary units. The detection cut-off was defined as 2 standard deviations below the mean healthy control values.

IFN signalling bioassay

To assess the ability of patient serum to block T1IFN and T3IFN pathway activation, HEK-293 cells were used which express luciferase, controlled by an *ISRE* (IFN-sensitive response element) sensitive to both T1IFN and T3IFN signalling.[25] HEK-293-*ISRE* cells were cultured at 37 °C 5% CO_2 in DMEM containing 10% FBS and 1% penicillin/streptomycin. Assays were performed in photometric 96 well-plates with cells seeded at a density of $2x10^5$ cells/ml. Serum was used with an end dilution of 1:10 in the presence of media (no IFN), 0.1 ng/ml IFNα2a (hereafter IFNα), 0.1 ng/ml IFNω, or 5 ng/ml IFNλ1 (hereafter IFNλ). The cells were incubated overnight, cells lysed, luciferase substrate added, and light units measured using a luminometer (BioTek Synergy H1 Multimode Reader). Samples were run in duplicate on different plates. IFN signalling activity was calculated from fold induction of samples over negative control (media only) and expressed as percentages normalised to activation by each IFN dose.

Statistical analyses

All analyses were performed using GraphPad Prism V10.0.2 or STATA. Mann-Whitney *U* test or Kruskal-Wallis test (for groups of ≥3) were used to compare unpaired data, while Wilcoxon matched-pairs sign rank test (Wilcoxon) was used to compare paired data. Fisher's exact test or Chi-squared test were used for contingency tables. Auto-Ab levels were log transformed as continuous dependent variables to fit a random intercepts model which accounted for clustering by individuals. Separate models were run for each auto-Ab. Given the exploratory nature of the study, univariate analysis was performed for *a priori* variables. For CHB1, missing data were interpreted as missing completely at random and no substitution or imputation methods were applied. p values <0.05 were considered statistically significant.

Ethics

Serum samples were accessed via RFL and UCL Biobank ethical Review Committee (UCL/RFL Biobank; REC reference: 11/WA/0077 or 21/WA/0388), Barts and The London NHS Trust Ethics Review Board (REC reference 10/H0715/39 or 16/LO/1699) and UCL systemic lupus erythematosus cohort study (REC reference no. 14/SC/1200). Informed consent was obtained from all participants.

Results

Overall, 16.7% (46/276) of patients with CHB showed evidence of any anti-IFN auto-Ab production, with 6.5% (18/276) demonstrating auto-Abs against all three IFN subtypes (Table 1; Fig. 1A). Compared to healthy controls, people with

Table 1. Overall anti-IFN auto-Ab outcomes including PegIFNα-exposed participants.

	Healthy controls	CHB1	CHB2	CHB total
Total	94	198	78	276
Anti-IFNα auto-Ab positive (n,%)	3 (3.2)	18 (9.1)	11 (14.1)	29 (10.5)
Median level (Gyros; 95% CI)	8 (6-10)	20 (18-22)	16 (15-21)	-
De novo during PegIFNα	-	-	7	-
Neutralising	0	0	1	1
Anti-IFNω auto-Ab positive	1 (1.1)	16 (8.1)	12 (15.3)	28 (10.1)
Median level	9 (8-11)	17 (15-19)	16 (14-22)	-
De novo during PegIFNα	-	-	7	-
Neutralising	0	2	0	2
Anti-IFNλ auto-Ab positive	1 (3.2)	24 (12.1)	15 (19.2)	39 (14.1)
Median level	15 (10-18)	21 (19-24)	17 (15-21)	-
De novo during PegIFNα	-	-	9	-
Neutralising	0	0	0	0
Any anti-T1IFN auto-Ab positive	2 (6.5)	21 (10.6)	18 (23.1)	39 (14.1)
De novo during PegIFNα	-	-	11	-
Neutralising	0	2	1	3
Any auto-Ab	2 (6.5)	25 (12.6)	21 (26.9)	46 (16.7)
De novo during PegIFNα	-	-	11	-
Neutralising	0	2	1	3

Auto-Abs, auto-antibodies; CHB, chronic hepatitis B; IFN, interferon; PegIFNα, pegylated-IFNα.

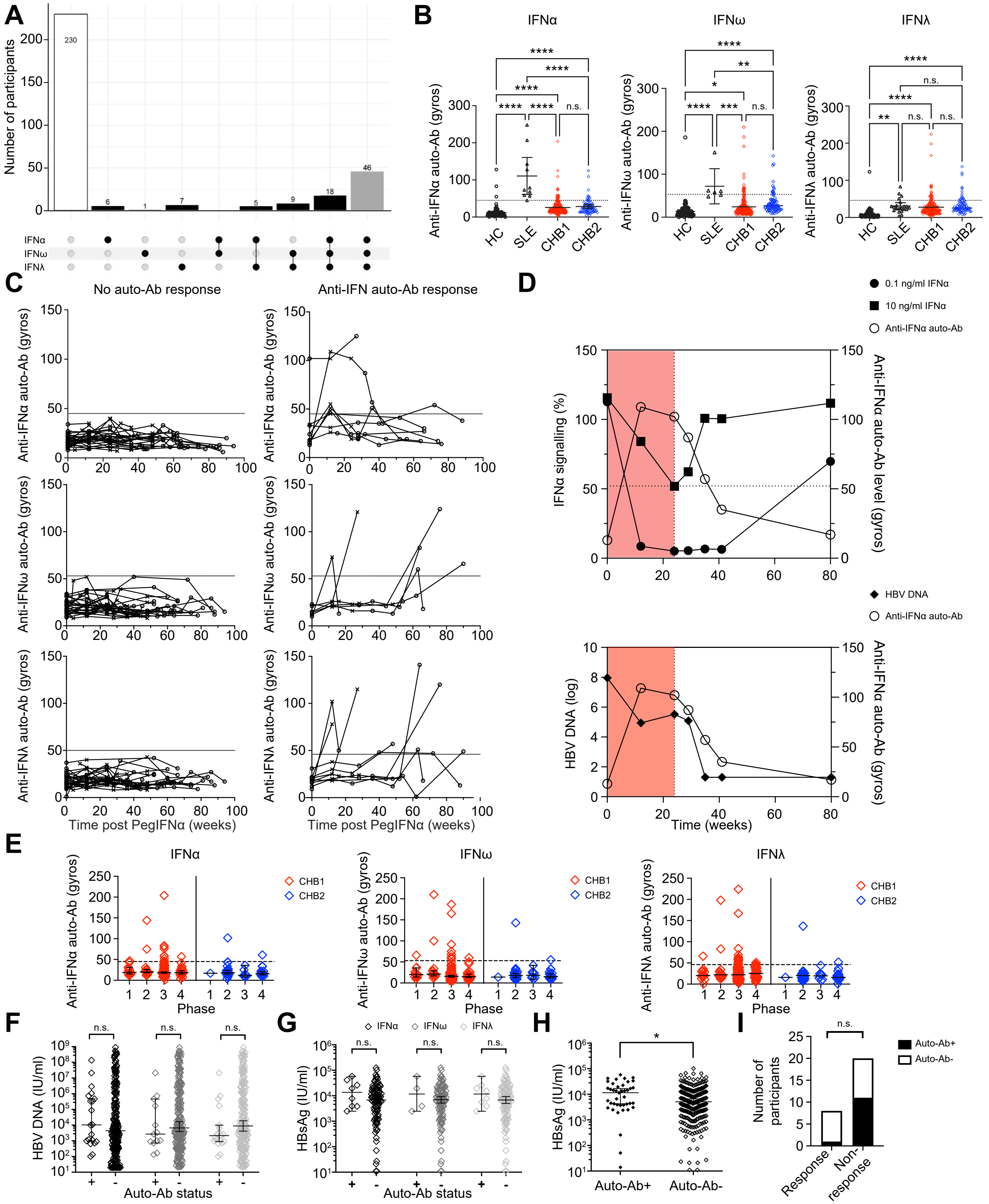

Fig. 1. Prevalence, function and clinical associations of auto-Abs against three subtypes of IFN. (A) Upset plot of number of participants with serum auto-Ab below (hollow bars) and above the level of detection (solid bars) measured by Gyros immunoassay, against three IFN subtypes with intersecting seroreactivity illustrated by upset plot connecting lines. Participants with any auto-Ab at any time are shown by the grey bar. (B) Scatter plot of serum auto-Ab levels against three IFN subtypes expressed as arbitrary units (Gyros). HC (healthy controls), SLE (patients with SLE and known auto-Ab status), CHB1, CHB2 (highest auto-Ab levels from

CHB from either cohort had a higher frequency of detectable auto-Abs against IFNα (10.5% *vs.* 3.2%, p = 0.0324; Table 1; Fig. 1B), IFNω (10.1% *vs*. 1.1%, p = 0.0030) and IFNλ (14.1% *vs.* 1.1%, p <0.0001). Median auto-Ab levels against all IFN subtypes were also significantly higher in both CHB cohorts compared to healthy controls (Fig. 1B).

For participants in CHB2 with serum samples available pre- and post-PegIFNα therapy, 20.0% (7/35) developed *de novo* anti-IFNα auto-Abs, 20.0% (7/35) anti-IFNω auto-Abs, and 25.7% (9/35) anti-IFNλ auto-Abs (Table 1; Fig. 1C). There were no clear differences in hepatology parameters prior to initiation of PegIFNα therapy between those who developed *de novo* auto-Abs compared to those who did not (Table S2). Individuals (n = 7) developing *de novo* anti-IFNα auto-Abs after PegIFNα treatment sero-reverted within 12 months and auto-Ab levels continued to increase post-PegIFNα therapy in one individual (Fig. 1C). Most individuals acquiring auto-Abs against IFNω and IFNλ remained seropositive throughout the course of follow-up (Fig. 1C).

We tested IFN neutralisation *in vitro* and showed this was only present in one individual with anti-IFNα auto-Abs (1/29, 3.4%; Fig. 1D), while receiving PegIFNα, and three individuals with anti-IFNω auto-Abs (3/28, 10.7%), one with and two without PegIFNα exposure (Table S1).

There was no difference in auto-Ab levels between CHB phases (Fig. 1E) and median levels of HBV DNA in treatment-naïve individuals did not significantly differ by auto-Ab status (Fig. 1F). Median levels of HBsAg were higher for individuals with detectable auto-Abs against each IFN subtype compared to individuals without auto-Abs, although these differences did not individually reach statistical significance (Fig. 1G). Overall median HBsAg levels were greater in patients with any detectable auto-Abs (11,466 IU/ml, 95% CI 4,236-16,655) compared to those without (5,076 IU/ml, 95% CI 4,119-6,609, p = 0.0110; Fig. 1H). A significant association between anti-IFNα auto-Abs and HBsAg levels was noted by random intercepts modelling (coefficient 0.11, 95% CI 0.02-0.19, p = 0.017; Table S3). Interestingly, all individuals with any detectable anti-T1FN auto-Abs demonstrated non-response to PegIFNα. All PegIFNα responders were seronegative for anti-T1IFN auto-Abs but one PegIFNα responder had detectable anti-IFNλ auto-Abs (Fig. 1I); however, this association within a small cohort did not reach statistical significance (p = 0.0882).

Discussion

In this study, we demonstrate the high prevalence of auto-Abs against T1 and T3IFNs in people living with CHB; these auto-Abs were associated with impaired control of HBV replication and non-response to PegIFNα treatment. We noted, in our study, that the incidence of developing anti-IFNα auto-Abs, induced by PegIFNα, was comparable to that seen with standard IFN prior to pegylation (7-39%).[17] Most participants with auto-Abs had reactivities against two or three IFN subtypes (32/46, 69.6%). Co-carriage of anti-IFNα and anti-IFNω auto-Abs is as high as 51% in COVID-19,[26,27] and anti-T3IFN auto-Ab prevalence has only been measured previously in two COVID-19 studies (3.6-10.1%)[28,29] and thus prior data in CHB remains limited; our study is thus key in filling this knowledge gap.

The prevalence of neutralising auto-Abs in our study (4/276, 1.4%) remained low, but was notably still greater than reported in the general population.[28,30] Most binding auto-Abs did not neutralise their target antigen *in vitro,* for which there may be various reasons. Potential insensitivity of the reporter assay is a possibility, although this has been used across other studies[30] or there may be alternative non-Fab-mediated effector functions of auto-Abs *in vivo*. Importantly for pathogenic anti-IFN γ auto-Abs, associated with severe mycobacterial and fungal infections, Fc-mediated antagonism of T1IFN signalling and innate cell cytotoxicity have been reported.[31] Thus, the effector function of auto-Abs detected in our study may not be Fab-mediated, potentially explaining the apparent association with increased virus replication.

Even in the absence of specific anti-IFNα auto-Abs, all individuals with any auto-Abs demonstrated PegIFNα non-response. Carriage of any auto-Ab was associated with higher median HBsAg levels, consistent with the hypothesis that these auto-Abs may antagonise antiviral IFN signalling, leading to compromised HBV control. Conversely, high HBsAg load may drive the development of anti-IFNα auto-Abs, due to the potential induction of IFNα during spontaneous hepatic flares[32] There is minimal structural homology between T1 and T3IFNs, which suggests that the presence of auto-Abs against multiple IFN subtypes, rather than evidence of cross-reactivity, represents broad breaches of immune tolerance as seen in COVID-19. PegIFNα therapy is also associated with *de novo* anti-thyroid auto-Abs.[33] Auto-Abs may therefore represent biomarkers reflecting an immune phenomenon predisposing to increased HBV replication that is IFN-refractory, such as increased frequency of atypical B cells which are associated with auto-Ab production and impaired anti-HBV immunity.[34,35]

The major limitation of our study is its retrospective design; thus, the sampling strategy was not designed to support definitive analyses for auto-Ab seroprevalence or associations with clinical outcomes. The relationship between HBsAg and auto-Ab is intriguing, however, we are unable to infer direction of cause or effect. Despite longitudinal sampling, auto-Abs were frequently only detected late in follow-up, which limits characterisation of the trajectory of anti-IFN auto-Ab production. Furthermore, due

longitudinal samples shown for CHB2). The cut-off for detection for each auto-Ab is shown by the dotted horizontal line. Median levels shown with error bars for 95% CIs. Analysed by Kruskall-Wallis ANOVA with Bonferroni correction. (C) Line charts for serum auto-Ab levels against three IFN subtypes in longitudinal CHB2 samples 2012-2016 for individuals with no and any auto-Ab positivity. Current PegIFNα treatment is indicated by cross plots. (D) IFNα signalling neutralisation by single participant longitudinal serum samples, with anti-IFNα auto-Ab levels (empty circle plots) plotted against 0.1 ng/ml IFNα (solid circle plots) and 10 ng/ml IFNα (square plots) signalling activation, or HBV DNA (log). Cut-off of detection for auto-Ab shown by horizontal dotted line. PegIFNα exposure shown by pink block prior to NUC therapy. (E) Auto-Ab levels against three IFN subtypes organised by CHB phase for CHB1 and CHB2 without PegIFNα exposure. (F) HBV DNA (IU/ml; n = 36 missing data) and (G) HBsAg levels (IU/ml; n = 44 missing data) for all individuals with CHB without PegIFNα exposure organised by auto-Ab status against three IFN subtypes. (H) HBsAg levels for all CHB (with or without PegIFNα exposure) organised into columns with or without any detectable auto-Ab. For (E-H), median levels shown with error bars for 95% CIs. Analysed by Mann-Whitney *U* test. (I) Frequency of auto-Abs by PegIFNα response and non-response. Analysed by Fisher's exact test. n.s. = not significant; *p <0.05, **p <0.05,****p <0.0001. Auto-Abs, auto-antibodies; CHB, chronic hepatitis B; HC, healthy control; IFN, interferon; NUC, nucleos(t)ide analogue; PegIFNα, pegylated-IFNα; SLE, systemic lupus erythematosus.

to the retrospective study design, we acknowledge some missing data, and although no CHB1 participants were receiving PegIFNα at the time of sample collection, we cannot exclude historical IFN therapy. Previous IFN treatment in some CHB1 participants may explain why overall auto-Ab profiles were comparable between the two cohorts.

In summary, our study suggests that anti-T1 and T3IFN auto-Abs are common in the UK CHB population. Our data can be used to design larger seroprevalence and immunophenotyping studies to characterise this and broader autoreactivity in CHB, which may be associated with treatment failure and have significant implications for future immune-mediated HBV therapies. PegIFNα-induced neutralising auto-Abs could be risk factors for life-threatening outcomes of acute respiratory virus infection and live-virus vaccinations in the CHB population which may require enhanced monitoring.

Affiliations

[1]*Infection and Immunity, University College London, London, UK;* [2]*Royal Free London NHS Foundation Trust, London, UK;* [3]*Institute for Global Health, University College London, London, UK;* [4]*Barts Liver Centre, Blizard Institute, Barts and The London, School of Medicine & Dentistry, Queen Mary University of London, London, UK*

Abbreviations

Auto-Abs, auto-antibodies; CHB, chronic hepatitis B; IFN, interferon; PegIFNα, pegylated-IFNα; T1IFN, type 1 IFN; T3IFN, type 3 IFN.

Financial support

This works was partly supported by a University College London (UCL) Therapeutic Acceleration Scheme Grant (553191.D-OTH.178973).

Conflicts of interest

MKM has received collaborative research funding from Gilead Sciences, F. Hoffmann-La Roche and Immunocore and has served as a consultant or on advisory boards for Gilead Sciences, F. Hoffmann-La Roche, Immunocore and GSK. Other authors have no conflicts of interest.

Please refer to the accompanying ICMJE disclosure forms for further details.

Authors' contributions

Conceived and designed study, performed some experiments and analysed data, and wrote the manuscript: DLF. Designed study, analysed data and wrote the manuscript: USG, MKM. Provided intellectual input and resources: HFB, MC, PTFK, LEM. Analysed data and performed statistical model analyses: DE. Performed experiments and analysed data: RH, OI, NK, OP, SG. Critically reviewed and approved the final manuscript: all authors.

Data availability statement

The data supporting the results of this study are available from the corresponding author upon reasonable request. The data are not publicly available due to privacy/ethical restrictions.

Supplementary data

Supplementary data to this article can be found online at https://doi.org/10.1016/j.jhepr.2025.101382.

References

[1] Cooke GS, Andrieux-Meyer I, Applegate TL, et al. Accelerating the elimination of viral hepatitis: a lancet gastroenterology & hepatology commission. Lancet Gastroenterol Hepatol 2019;4:135–184. https://doi.org/10.1016/S2468-1253(18)30270-X.
[2] Peters MG, Yuen MF, Terrault N, et al. Chronic hepatitis B finite treatment: similar and different concerns with new drug classes. Clin Infect Dis 2024;78:983. https://doi.org/10.1093/CID/CIAD506.
[3] Maini MK, Burton AR. Restoring, releasing or replacing adaptive immunity in chronic hepatitis B. Nat Rev Gastroenterol Hepatol 2019;16(11):662–675. https://doi.org/10.1038/s41575-019-0196-9. 2019;16.
[4] Maini MK, Gehring AJ. The role of innate immunity in the immunopathology and treatment of HBV infection. J Hepatol 2016;64:S60–S70. https://doi.org/10.1016/J.JHEP.2016.01.028.
[5] McNab F, Mayer-Barber K, Sher A, et al. Type I interferons in infectious disease. Nat Rev Immunol 2015;15:87–103. https://doi.org/10.1038/nri3787.
[6] Wack A, Terczyńska-Dyla E, Hartmann R. Guarding the frontiers: the biology of type III interferons. Nat Immunol 2015;16:802. https://doi.org/10.1038/NI.3212.
[7] Dunn C, Peppa D, Khanna P, et al. Temporal analysis of early immune responses in patients with acute hepatitis B virus infection. Gastroenterology 2009;137:1289–1300. https://doi.org/10.1053/J.GASTRO.2009.06.054.
[8] Fletcher SP, Chin DJ, Ji Y, et al. Transcriptomic analysis of the woodchuck model of chronic hepatitis B. Hepatology 2012;56:820–830. https://doi.org/10.1002/HEP.25730.
[9] Stacey AR, Norris PJ, Qin L, et al. Induction of a striking systemic cytokine cascade prior to peak viremia in acute human immunodeficiency virus type 1 infection, in contrast to more modest and delayed responses in acute hepatitis B and C virus infections. J Virol 2009;83:3719–3733. https://doi.org/10.1128/JVI.01844-08.
[10] De Groen RA, McPhee F, Friborg J, et al. Endogenous IFNλ in viral hepatitis patients. J Interferon Cytokine Res 2014;34:552–556. https://doi.org/10.1089/JIR.2013.0068.
[11] Montanari NR, Ramírez R, Aggarwal A, et al. Multi-parametric analysis of human livers reveals variation in intrahepatic inflammation across phases of chronic hepatitis B infection. J Hepatol 2022;77:332–343. https://doi.org/10.1016/j.jhep.2022.02.016.
[12] Narmada BC, Khakpoor A, Shirgaonkar N, et al. Single-cell landscape of functionally cured chronic hepatitis B patients reveals activation of innate and altered CD4-CTL-driven adaptive immunity. J Hepatol 2024;81:42–61. https://doi.org/10.1016/J.JHEP.2024.02.017/ATTACHMENT/31EB8652-62C6-49B6-9E91-104D97E8EA3C/MMC3.PDF.
[13] van Buuren N, Ramirez R, Turner S, et al. Characterization of the liver immune microenvironment in liver biopsies from patients with chronic HBV infection. JHEP Rep 2022;4:100388. https://doi.org/10.1016/J.JHEPR.2021.100388.
[14] Pagliaccetti NE, Chu EN, Bolen CR, et al. Lambda and alpha interferons inhibit hepatitis B virus replication through a common molecular mechanism but with different in vivo activities. Virology 2010;401:197–206. https://doi.org/10.1016/J.VIROL.2010.02.022.
[15] Cao Y, Zhang R, Zhang W, et al. IL-27, a cytokine, and IFN-λ1, a type III IFN, are coordinated to regulate virus replication through type I IFN. J Immunol 2014;192:691–703. https://doi.org/10.4049/JIMMUNOL.1300252.
[16] Bastard P, Gervais A, Le Voyer T, et al. Human autoantibodies neutralizing type I IFNs: from 1981 to 2023. Immunol Rev 2024;322:98–112. https://doi.org/10.1111/IMR.13304.
[17] Ye J, Chen J. Interferon and hepatitis B: current and future perspectives. Front Immunol 2021;12. https://doi.org/10.3389/FIMMU.2021.733364.
[18] Arends P, Van Der Eijk AA, Sonneveld MJ, et al. Presence of anti-interferon antibodies is not associated with non-response to pegylated interferon treatment in chronic hepatitis B. Antivir Ther 2014;19:423–427. https://doi.org/10.3851/IMP2711.
[19] Tan G, Song H, Xu F, et al. When hepatitis B virus meets interferons. Front Microbiol 2018;9. https://doi.org/10.3389/FMICB.2018.01611.
[20] Nishio A, Bolte FJ, Takeda K, et al. Clearance of pegylated interferon by Kupffer cells limits NK cell activation and therapy response of patients with HBV infection. Sci Transl Med 2021;13. https://doi.org/10.1126/SCITRANSLMED.ABA6322.
[21] Etzion O, Hamid S, Lurie Y, et al. Treatment of chronic hepatitis D with peginterferon lambda-the phase 2 LIMT-1 clinical trial. Hepatology 2023;77:2093–2103. https://doi.org/10.1097/HEP.0000000000000309.
[22] European Association for the Study of the Liver. EASL 2017 Clinical Practice Guidelines on the management of hepatitis B virus infection. J Hepatol 2017;67:370–398. https://doi.org/10.1016/J.JHEP.2017.03.021.
[23] Bradford HF, Haljasmägi L, Menon M, et al. Inactive disease in patients with lupus is linked to autoantibodies to type I interferons that normalize blood IFNα and B cell subsets. Cell Rep Med 2023;4:100894. https://doi.org/10.1016/J.XCRM.2022.100894.

[24] Bastard P, Gervais A, Voyer T Le, et al. Autoantibodies neutralizing type I IFNs are present in ∼4% of uninfected individuals over 70 years old and account for ∼20% of COVID-19 deaths. Sci Immunol 2021;6:4340–4359. https://doi.org/10.1126.
[25] Fink DL, Idilli O, Shields A, et al. Prevalence of anti-interferon-alpha autoantibodies in patients with antibody deficiency. J Clin Immunol 2024;44(7):1–4. https://doi.org/10.1007/S10875-024-01761-2. 2024;44.
[26] Bastard P, Gervais A, Voyer T Le, et al. Autoantibodies neutralizing type I IFNs are present in ∼4% of uninfected individuals over 70 years old and account for ∼20% of COVID-19 deaths. Sci Immunol 2021;6:4340–4359. https://doi.org/10.1126.
[27] Bastard P, Rosen LB, Zhang Q, et al. Autoantibodies against type I IFNs in patients with life-threatening COVID-19. Science 2020;370. https://doi.org/10.1126/SCIENCE.ABD4585.
[28] Vanker M, Särekannu K, Fekkar A, et al. Autoantibodies neutralizing type III interferons are uncommon in patients with severe coronavirus disease 2019 pneumonia. J Interferon Cytokine Res 2023;43:379–393. https://doi.org/10.1089/JIR.2023.0003.
[29] Credle JJ, Gunn J, Sangkhapreecha P, et al. Unbiased discovery of autoantibodies associated with severe COVID-19 via genome-scale self-assembled DNA-barcoded protein libraries. Nat Biomed Eng 2022;6(8):992–1003. https://doi.org/10.1038/s41551-022-00925-y. 2022;6.
[30] Bastard P, Gervais A, Voyer T Le, et al. Autoantibodies neutralizing type I IFNs are present in ∼4% of uninfected individuals over 70 years old and account for ∼20% of COVID-19 deaths. Sci Immunol 2021;6:4340–4359. https://doi.org/10.1126.
[31] Shih HP, Ding JY, Bellón JS, et al. Pathogenic autoantibodies to IFN-γ act through the impedance of receptor assembly and Fc-mediated response. J Exp Med 2022;219. https://doi.org/10.1084/JEM.20212126.
[32] Dunn C, Brunetto M, Reynolds G, et al. Cytokines induced during chronic hepatitis B virus infection promote a pathway for NK cell-mediated liver damage. J Exp Med 2007;204:667–680. https://doi.org/10.1084/JEM.20061287.
[33] Liu Y, Zheng Y, Lin X, et al. Analysis of clinical characteristics of thyroid disorders in patients with chronic hepatitis B treated with pegylated-interferon alpha. BMC Endocr Disord 2023;23. https://doi.org/10.1186/S12902-023-01371-W.
[34] Cancro MP. Age-associated B cells. Annu Rev Immunol 2020;38:315–340. https://doi.org/10.1146/ANNUREV-IMMUNOL-092419-031130.
[35] Burton AR, Pallett LJ, McCoy LE, et al. Circulating and intrahepatic antiviral B cells are defective in hepatitis B. J Clin Invest 2018;128:4588–4603. https://doi.org/10.1172/JCI121960.

Keywords: chronic hepatitis B; CHB; interferon; auto-antibodies.

Received 4 October 2024; received in revised form 9 February 2025; accepted 24 February 2025; Available online 28 February 2025

Journal of Hepatology, Volume *7*

Supplemental information

Auto-antibodies against interferons are common in people living with chronic hepatitis B virus infection and associate with PegIFNα non-response

Douglas L. Fink, David Etoori, Robert Hill, Orest Idilli, Nikita Kartikapallil, Olivia Payne, Sarah Griffith, Hannah F. Bradford, Claudia Mauri, Patrick T.F. Kennedy, Laura E. McCoy, Mala K. Maini, and Upkar S. Gill

Auto-antibodies against interferons are common in people living with chronic hepatitis B virus infection and associate with PegIFNα non-response

Douglas L. Fink, David Etoori, Robert Hill, Orest Idilli, Nikita Kartikapallil, Olivia Payne, Sarah Griffith, Hannah F. Bradford, Claudia Mauri, Patrick T.F. Kennedy, Laura E. McCoy, Mala K. Maini, Upkar S. Gill

Table S1……………………………………………………………………………2

Table S2……………………………………………………………………………3

Table S3……………………………………………………………………………4

	HC	CHB1	CHB2	p value
Total	94	198	78	
Age (IQR)	40 (31-64)	48 (41-54)	32 (28-41)	0.0001
Sex<br>Male<br>Female<br>Missing	 47 (50.0)<br>44 (46.8)<br>3 (3.2)	 102 (51.5)<br>75 (37.9)<br>21 (10.6)	-	-
HBeAg<br>Positive<br>Negative<br>Missing	-	 30 (15.2)<br>139 (70.2)<br>29 (14.6)	 36 (46.1)<br>41 (52.6)<br>1 (1.3)	<0.001
HBV DNA<br>Undetectable<br>Detectable<br>Missing	-	 29 (14.6)<br>134 (67.7)<br>35 (17.7)	 6 (7.7)<br>71 (91.0)<br>1 (1.3)	0.04
Median log HBV DNA (IU/ml, IQR)	-	3.17 (2.00-4.14)	6.07 (3.77-7.54)	<0.001
Median log HBsAg (IU, IQR)<br>Missing	-	3.59 (3.08-4.27)<br>43 (21.7)	3.91 (3.52-4.19)<br>1 (1.3)	0.1229
Median ALT (IU/L, IQR)<br>Missing	-	33 (23-48)<br>34 (17.2)	65 (35-129)<br>1 (1.3)	<0.001
CHB phase<br>1<br>2<br>3<br>4<br>5<br>Unknown	-	 14 (7.1)<br>15 (7.6)<br>108 (54.5)<br>26 (13.1)<br>2 (1.0)<br>33 (16.7)	 8 (10.3)<br>28 (35.9)<br>14 (17.9)<br>27 (34.6)<br>0<br>1 (1.3)	<0.001
PegIFNα exposure<br>Samples pre and post PegIFNα	-	0<br>0	58 (74.3)<br>35 (44.8)	-
Nucleoside treatment during study	-	26 (13.1)	35 (44.8)	<0.001

Table S1. Cohort clinical characteristics.

HC= healthy controls ; CHB1= chronic hepatitis B virus infection cohort 1; CHB2= chronic hepatitis B virus cohort 2 (without PegIFNα exposure); IQR=inter-quartile range; ALT=alanine transaminase. Continuous variables reported for first available sample for CHB2 cohort with longitudinal sampling. Comparisons of median values by Mann-Whitney U-test or Kruskal-Wallis test (for groups of 3 or more); comparisons of frequency by Chi-squared test or Fisher's exact test (for groups of 5 or less). $p<0.05$ = statistically significant.

	No auto-Ab	Anti-IFNα auto-Ab	Anti-IFNω auto-Ab	Anti-IFNλ auto-Ab	P value
Total<br>Auto-Ab pre PegIFNα<br>Auto-Ab post PegIFNα	22 (61.1)<br>-<br>-	8 (22.2)<br>1<br>7	7 (19.4)<br>0<br>7	9 (25.0)<br>0<br>9	-
Age (IQR)	34 (29-43)	35 (29-43)	38 (29-45)	35 (28-45)	0.7256
HBeAg<br>Positive<br>Negative<br>Missing	 10 (45.5)<br>12<br>0	 4 (50.0)<br>4<br>0	 1 (14.3)<br>5<br>1	 3<br>6<br>0	0.6024
Median log HBV DNA pre PegIFNα (IU/ml, IQR)	6.6 (6.1-7.7)	8.1 (6.4-8.6)	6.4 (4.6-8.1)	6.9 (4.8-8.1)	0.2799
Median log HBsAg pre PegIFNα (IU, IQR)	3.9 (3.8-4.2)	4.0 (3.1-4.6)	4.1 (3.9-4.3)	4.0 (3.8-4.3)	0.5144
Median ALT pre PegIFNα (IU/L, IQR)	114 (83-256)	164 (73-210)	97 (57-210)	91 (61-199)	0.8743
Median Ishak liver fibrosis score (IQR)	3.0 (1.0-4.0)	3.0 (1.0-3.5)	1.5 (1.0-2.5)	2.0 (1.0-3.0)	0.6693
Median HAI score (IQR)	5.0 (4.0-8.0)	6.0 (3.5-7.0)	5.0 (3.5-6.3)	5.0 (4.0-7.0)	0.9285
HBV genotype<br>A<br>B<br>C<br>D<br>E<br>Missing	 2<br>1<br>4<br>8<br>6<br>2	 1<br>0<br>1<br>3<br>1<br>1	 1<br>0<br>0<br>1<br>2<br>3	 0<br>0<br>1<br>1<br>3<br>3	0.7390

Table S2. Clinical characteristics of CHB2 PegIFNα-exposed individuals with serum samples pre and post PegIFNα

HC= healthy controls ; CHB1= chronic hepatitis B virus infection cohort 1; CHB2= chronic hepatitis B virus cohort 2 (without PegIFNα exposure); IQR=inter-quartile range; ALT=alanine transaminase; HAI=Histological Activity Index. Comparisons of median values by Mann-Whitney U-test or Kruskal-Wallis test (for groups of 3 or more); comparisons of frequency by Chi-squared test or Fisher's exact test (for groups of 5 or less). $p<0.05$ = statistically significant.

		IFNα		IFNω		IFNλ	
Total	513						
	Median [IQR]	Coefficient (95% CI)	p-value	Coefficient (95% CI)	p-value	Coefficient (95% CI)	p-value
log(HBsAg)	3.73 [3.37, 4.14]	**0.11 (0.02, 0.19)**	**0.017**	0.02 (-0.07, 0.11)	0.64	0.02 (-0.07, 0.12)	0.606
log(HBVDNA)	3.08 [1.46, 5.19]	0.01 (-0.01, 0.03)	0.181	0.01 (-0.01, 0.03)	0.429	-0.003 (-0.04, 0.02)	0.805
log(ALT)	1.59 [1.40, 1.85]	0.03 (-0.07, 0.12)	0.566	-0.003 (-0.12, 0.11)	0.958	-0.06 (-0.19, 0.07)	0.366
	n (%)						
Age							
16-24	25 (4.9)	-0.02 (-0.32, 0.27)	0.876	-0.06 (-0.39, 0.28)	0.743	**-0.42 (-0.77, -0.08)**	**0.017**
25-34	161 (31.4)	Reference	—	Reference	—	Reference	—
35-44	150 (29.2)	0.03 (-0.13, 0.19)	0.721	0.002 (-0.17, 0.17)	0.983	0.07 (-0.10, 0.24)	0.432
45-54	117 (22.8)	0.13 (-0.05, 0.31)	0.159	0.17 (-0.01, 0.35)	0.063	0.17 (-0.02, 0.35)	0.077
55+	57 (11.1)	0.04 (-0.16, 0.25)	0.676	-0.03 (-0.24, 0.18)	0.79	0.09 (-0.13, 0.30)	0.42
HBeAg							
Negative	328 (63.9)	Reference	—	Reference	—	Reference	—
Positive	148 (28.9)	0.07 (-0.06, 0.20)	0.278	0.12 (-0.02, 0.26)	0.09	0.01 (-0.13, 0.16)	0.864
Missing	37 (7.2)	—	—	—	—	—	—
IFN therapy at time of sample							
No IFN exposure	268 (52.2)	Reference	—	Reference	—	Reference	—
Current IFN exposure	81 (15.8)	**0.17 (0.06, 0.27)**	**0.002**	**0.14 (0.01, 0.27)**	**0.031**	0.12 (-0.02, 0.27)	0.098
Post IFN exposure	158 (30.8)	0.01 (-0.09, 0.11)	0.83	**0.16 (0.04, 0.28)**	**0.011**	0.09 (-0.04, 0.23)	0.161

Table S3. Random intercepts model for auto-Ab levels

Random intercepts model for log transformed auto-Ab levels against IFN sub-types organised by univariate analyses for all available CHB samples from both cohorts including longitudinal samples (n=513). Significant interactions are highlighted in bold where $p < 0.05$.
